# Developing and Validating a Tablet Version of an Illness Explanatory Model Interview for a Public Health Survey in Pune, India

**DOI:** 10.1371/journal.pone.0107374

**Published:** 2014-09-18

**Authors:** Joseph G. Giduthuri, Nicolas Maire, Saju Joseph, Abhay Kudale, Christian Schaetti, Neisha Sundaram, Christian Schindler, Mitchell G. Weiss

**Affiliations:** 1 Department of Epidemiology and Public Health, Swiss Tropical and Public Health Institute, Basel, Switzerland; 2 University of Basel, Basel, Switzerland; 3 The Maharashtra Association of Anthropological Sciences, Centre for Health Research and Development, Pune, India; Centers for Disease Control, Taiwan

## Abstract

**Background:**

Mobile electronic devices are replacing paper-based instruments and questionnaires for epidemiological and public health research. The elimination of a data-entry step after an interview is a notable advantage over paper, saving investigator time, decreasing the time lags in managing and analyzing data, and potentially improving the data quality by removing the error-prone data-entry step. Research has not yet provided adequate evidence, however, to substantiate the claim of fewer errors for computerized interviews.

**Methodology:**

We developed an Android-based illness explanatory interview for influenza vaccine acceptance and tested the instrument in a field study in Pune, India, for feasibility and acceptability. Error rates for tablet and paper were compared with reference to the voice recording of the interview as gold standard to assess discrepancies. We also examined the preference of interviewers for the classical paper-based or the electronic version of the interview and compared the costs of research with both data collection devices.

**Results:**

In 95 interviews with household respondents, total error rates with paper and tablet devices were nearly the same (2.01% and 1.99% respectively). Most interviewers indicated no preference for a particular device; but those with a preference opted for tablets. The initial investment in tablet-based interviews was higher compared to paper, while the recurring costs per interview were lower with the use of tablets.

**Conclusion:**

An Android-based tablet version of a complex interview was developed and successfully validated. Advantages were not compromised by increased errors, and field research assistants with a preference preferred the Android device. Use of tablets may be more costly than paper for small samples and less costly for large studies.

## Background

Paper-based questionnaires and interviews have long been standard tools for conducting socio-cultural, household, clinical and healthcare surveys. The popularity of advanced mobile devices (tablet computers) has increased dramatically in recent years, and they are rapidly embedding into the fabric of epidemiological and public health research. In many studies, these devices are replacing classical paper-based instruments. Since they were first used in epidemiological research surveys in the 1980s [1], electronic handheld devices, such as personal digital assistants (PDA) [2]–[3], mobile phones [4]–[6] and tablet computers [7]–[8] have become attractive tools for public health research because of notable advantages over paper tools.

Computer devices overcome significant limitations of paper-based interviews. They make field data more quickly available for analysis and review, and they may reduce errors by eliminating the data-entry step after initially entering the data during the interview. Electronic devices also enable implementation of quality control measures, such as range checks and skip logic navigation at an early stage in the process of acquiring, managing and analyzing data [4], [5], [8]. Personal digital assistants and cell phones already had advantages for collecting data, and as the technology has developed further, more advanced devices, i.e., smartphones and tablet computers, have replaced the older devices. Additional benefits of the newer devices beyond speed, image quality and better software include capacity for recording audio, cameras, and global positioning systems (GPS). Media files (pictures, audio and video) and exact geo-location of households may also be more easily included in data sets.

Comparative studies to validate mobile devices against paper-based instruments have mainly been conducted in clinical trials, and for patient diaries and patient-reported outcome studies in hospitals [2]. A few studies have considered the validity of advanced mobile devices that are now available for epidemiological field-based research [3]–[6]. None of these studies, however, fulfilled all desired criteria, namely, survey conducted in a community setting; direct comparison with reference to a gold standard; and consideration of an up-to-date, widely used and readily available technology. Validation studies require a research design that enables efficient identification of discrepancies in data sets derived from paper- and tablet-based interviews and an authoritative reference standard to assign errors when such discrepancies are identified. Maintaining qualitative narratives poses an additional challenge. Our study was designed to address such challenges in validating tablet devices, to determine whether the known advantages of removing a data-entry step are compromised by problems in the quality, user preferences and costs of tablet-based data collection for field-based interviews.

Experience of our research team in a recent study in Pune, India [9], provided an opportunity to adapt a complex paper-based interview with various field types and a navigation determined by skip-logic responses to designated questions. This interview for research in cultural epidemiology is based on the framework of the Explanatory Model Interview Catalogue (EMIC) [10]–[11] and had been developed for a post-pandemic study of influenza vaccine acceptance [9]. The EMIC interview benefited from prior experience in other studies of vaccine acceptance [12]–[15]. The availability of a research team experienced in its use provided an opportunity to compare the tablet and paper versions of the interview.

The broad aim of the study was to develop and validate a version of a complex interview with various field types for an Android device, using open-source Open Data Kit (ODK) software [16]. Specific aims included (i) analysis of discrepancies to determine whether the benefits of tablet computing compromise validity with more errors, (ii) assessment of the preferences of interviewers for the tablet or paper-based device, and (iii) comparison of the device-specific costs of the two options.

## Methods

### Setting

The EMIC interview used in the study was initially developed in a partnership between the Swiss Tropical and Public Health Institute (Swiss TPH), Basel, and the Maharashtra Association for the Anthropological Sciences (MAAS), Pune [Ref study protocol]. Development of the ODK version of the tablet-based EMIC interview was guided and supervised by the public health computing group at the Swiss TPH, which also supported a secure local host server for uploading data.

The study was planned in urban and rural localities of Pune district in Maharashtra, India. Urban interviews were conducted with household residents of Janata Vasahat, a large slum in Pune city. Rural interviews were conducted in three villages (viz., Gahunje, Salumbre, and Darumbre) in Mawal Tehsil, a sub-district of rural Pune district. Both urban and rural sites had been substantially affected during the influenza pandemic of 2009 [17].

### Instruments

The paper form and the ODK version of the interview for the tablet computer had an identical structure and data input fields to enable comparison. Inclusion of various types of questions in the complex interview provided an opportunity to acquire experience in adapting these questions from paper to Android-based interviews, and to examine the validity of different field types; e.g., integer codes, selection of ‘one option only,’ selection of ‘all that apply’ among multiple options, and open questions for either short or extended narrative responses.

#### Adaption of EMIC interview instrument

The interview adapted for this study was based on an abridged version of the paper-based EMIC interview that had previously been used in the completed cultural epidemiological field study of pandemic influenza vaccine acceptance and use [9]. Sections of this instrument queried social and demographic characteristics of respondents; priority symptoms, perceived causes and preferred help seeking for pandemic influenza based on a vignette depicting typical features of the illness, and questions about experience and preference for use of vaccines that were available in the pandemic at various levels of cost.

#### Development of an ODK form

The tablet version of this EMIC interview was created with ODK, and the ODK forms were created using the XLS Form design, making use of features such as range constraints and skip logic, which are explained in the online documentation for ODK [18]. The ODK Collect application was installed on the tablet computers, and the interview form was downloaded from the ODK Aggregate server. Tablet devices used in our study were an Android-based Samsung Galaxy Note 10.1. The instruments and comparative design were pre-tested in another area distinct from the study sites. Errors identified in pilot testing were corrected to ensure consistency for comparison of the paper and ODK interview forms.

### Training of interviewers

At the outset, four interviewers were selected, each with a Master’s degree and experience working with paper-based EMIC interviews. Two of the four had previously worked with the paper interview used in the prior study of influenza vaccine acceptance, and the other two had worked with a comparable paper instrument in another cultural epidemiological study conducted by MAAS on the stigma of leprosy. The four interviewers worked in two teams (A and B) of two researchers. Field work was planned to proceed sequentially at the urban site first and then at the rural site. A third team (C) of two interviewers with similar academic training replaced team A for the rural field work. Although experienced in other sociological surveys, team-C field research assistants had no prior experience with EMIC interviews, and they were trained before proceeding with their field work in this study. Once familiar with the structure and coding of paper-based EMIC interviews, all interviewers participated in three days of intensive training on the use of the tablet. Their training culminated in 12 pilot interviews before proceeding to study interviews.

### Study design

Each interview was conducted with two field research assistants (male and female), one who administered the interview and entered data using either a tablet device (T) or a paper form (P) form; a second researcher only entered data on the other device or paper form. The role of the field research assistant who administered the interview and entered the data was designated “lead” (L), and the role of the second interviewer who only entered data was designated “follow” (F) for that interview. The roles of field research assistants changed for each interview according to a designated cycle, and the paper and tablet devices were exchanged between the interviewers after two interviews. Each assistant was therefore scheduled to conduct equal numbers of T and P interviews, and to work in the role of L or F (Table 1). All interviews were audio-recorded with an external digital recorder, providing an authoritative reference to resolve discrepancies identified in analysis and to attribute errors to either the paper or tablet device.

**Table 1:** Four recurring roles for each of the field research assistants over the course of the study.

Interview number	Interviewer-1	Interviewer-2
1	PL	TF
2	PF	TL
3	TL	PF
4	TF	PL

*This cycle of respective roles in each interview repeats for the two field research assistants on each team using paper (P) or tablet (T) device, and functioning as interviewer (Lead, L) or follower (coder only, F).*

To assess the subjective preferences of the field research assistants for both devices in their respective roles as L or F, each field researcher completed a debriefing questionnaire after every interview. The questionnaire comprises questions about preference and problems encountered with the T or P device used in that interview by the field research assistant.

### Data collection

A total of 98 interviews were planned. Eligible respondents were 18 to 65 years of age, fluent in Marathi, and both mentally and physically able to complete the interview. Sampling maintained an equal balance of men and of women in both younger (18–45 years) and older (46–65) age groups. Households were randomly selected in the study communities, and respondents received information about the study and signed informed consent prior to their interview.

### Data management and security

Completed paper data forms were entered in Epi Info software (Version 3.5.4, CDC, Atlanta, GA, USA). Data cleaning for entry errors in the paper forms involved double entry to identify discrepancies, which were resolved by consulting the paper form (but not audio). For tablet data, field research assistants reviewed their entries and gave the tablets to the supervisor to verify completeness and upload data for analysis. The data were encrypted and uploaded over a Wi-Fi connection to a central server after returning to the office.

Data were cleaned to correct artifacts from the Epi Info and ODK entry to ensure appropriate matching in the structure of the two data sets. This procedure involved renaming some variables, formatting date and time fields, and processing short text fields for consistency of capitalization and deletion of white space to minimize insignificant pseudo-discrepancies. A python program [19] (Python V2.7.3, Python Foundation) was used to generate a discrepancy report for matched fields of the paper- and tablet-derived data sets.

### Ethical statement

Signed informed consent from respondents was obtained after study interests were explained to respondents, and confidentiality and anonymity were assured.. Participants were given a chance to withdraw from the study at any time. Interview content concerning the cultural epidemiology of pandemic influenza and the acceptability of vaccines at various levels of cost had been reviewed and approved by the Institutional Ethics Committee (IEC) of MAAS, and by the Ethics Commission of Basel (EKBB) for the Swiss TPH. These ethics committees were advised of additional interviews to develop an Android version of the explanatory model interview in an extension of the original study, and both acknowledged and accepted this amendment–IEC on 14 Jan 2013, reference: MAAS-IEC/2013/001; and EKBB on 14 Dec 2012, reference: 383/11.

## Analysis

### Discrepancy analysis

Matching fields of the paper and tablet datasets were compared to identify discrepancies, and a report of paper-tablet discrepancies was produced in an Excel spreadsheet. The total number of data fields per interview that were compared was 234. Each screen swipe also produced a time field indicating the time, which facilitated location of the immediately preceding question on the audio recording for verification of any identified discrepancies. Such discrepancies were listed on a worksheet with their item number and elapsed time in the interview to facilitate access to the authoritative audio recording to determine attribution of the error. Discrepancies must be explainable either by classifying each as an error in the use of one of the two devices only, or by errors in the use of both devices. If the audio could not clarify attribution of the error to one of the two devices, it was classified as unexplained. For a blinded assessment to avoid device-related bias in attributing errors, the list of discrepancies for resolution was prepared without indicating which of the discrepant responses were from tablet or paper. The researcher listening to the audio therefore did not know which discrepant response was associated with one or the other device. Subsequently, each discrepancy could then be classified as a paper error, tablet error, both paper and tablet error if both were incorrectly coded, or ambiguous if the audio did not enable distinguishing correct and incorrect coding (Table 2).

**Table 2:** Classification of discrepancies with reference to device attributable errors.

Type of error	Description of error
Paper	Paper entry incorrect
	Paper entry missing
	Tablet entry missing because paper interviewer (lead) did not follow the skip logic
Tablet	Tablet entry incorrect
	Tablet entry missing
	Paper entry missing because of tablet interviewer (lead) skip logic
Paper and tablet	Both paper and tablet entries incorrect or missing
Device non-specific	Recording inadequate to specify correct entry code (Ambiguous response or unclear audio)

The mean number of discrepancies and the device-specific attribution of errors were analyzed. Field research teams and residency status of respondents were compared to check for any significant difference in discrepancies with different interviewing devices. A mixed binomial regression model was used for analysis of device-specific error rates, adjusting for the influence of role in the interview (L or F), urban/rural location, interviewer and respondent characteristics (e.g., age, sex and education status) while treating respondent as random effect. Stata software (version 12.1, StataCorp, TX, USA) was used for the analysis.

### Subjective preference of interviewers

For each interview, both field research assistants completed a questionnaire asking about experience with the device used, its value and problems, and inquiring about any preference for one or the other in that interview. The frequency of device preferences was tabulated and a qualitative account was provided of difficulties encountered by the field research assistants.

### Cost comparison

To compare device-specific study costs, we considered the cost of tablet computers and server charges for a tablet-based and printing and data-entry expenses for a paper-based interview study. Other costs unrelated to devices were not included (e.g., preparation of the structure and content of the survey instrument, interviewer costs, field-study logistics and training). Basic infrastructural costs of computers and printers were not included in the comparison. As the time taken to carry out an interview was constrained by design to be identical for the two instruments, the cost of interviewers was omitted from the analysis. The cost analysis was based on the number of interviews in this study and extrapolated for larger samples, up to 1,000. To account for the interest in timely completion of larger studies, we considered the addition of one more study team for studies greater than 400 persons, and in further increments of 200. This required purchase of an additional tablet for an additional team but no incremental capital costs for an additional team using paper interviews.

## Results

Data collection was completed between February and April 2013. Of the potential respondents approached in 98 households, 2 refused and 96 agreed to be interviewed. Team-A completed 24 interviews in the urban site, and team C completed 24 interviews in the rural site. Team B completed 24 interviews each in the urban and rural sites. All interviews were in Marathi. We analysed 95 of the 96 interviews, because one urban interview was inadvertently deleted on the tablet device in an early interview.

The average time per interview for double entry of data from the paper form was approximately 30 minutes. Double-entry discrepancies for the paper forms were found in 92 interviews with a mean of 5.83 (2.49% of 234 fields compared) per interview for 95 interviews and analyzed, and they were resolved with reference to the paper hard copy, but without consulting the audio recording, to produce a paper-derived data set for comparison with the tablet-derived data set.

### Discrepancy analysis

The mean number of tablet-paper discrepancies per interview was 11.14, which is 4.76% of the 234 comparison fields of the interview in the data set. The mean number of paper-attributable errors was 4.68 (2.01%); for tablet-attributable errors the mean was 4.65 (1.99%). Discrepancy rates differed among field-research teams, and team C, which was the least experienced, had the highest rate (Table 3). Team C had the highest rates of both tablet errors and paper errors. Rates of tablet errors were significantly different across teams but rates of paper errors were not.

**Table 3:** Paper-tablet discrepancies and device attributable coding errors for the field research assistant teams.

Discrepancies & Type of error	Team-A (n = 24)		Team-B (n = 47)		Team-C (n = 24)		p-value
	Mean	%	Mean	%	Mean	%	
Discrepancies	10.50	4.49	9.26	3.96	15.46	6.61	<0.0001
Paper errors	4.29	1.84	4.09	1.75	6.25	2.67	0.009
Tablet errors	4.96	2.21	3.66	1.56	6.29	2.69	0.0004
Both paper and tablet errors	0.58	0.25	0.26	2.69	0.62	0.26	0.07
Unattributable errors	0.67	0.29	1.25	0.26	2.30	0.98	NA

*n: number of interviews.*

*%: Percentage of errors with reference to 234 comparison fields. p-value: Simple mixed binomial regression model with factor urban and rural.*

*NA: Not applicable.*

*The difference between the means of paper errors and tablet errors for each field-research team is statistically insignificant. Paired t-test p-values for team A, B and C are 0.50, 0.45 and 0.97 respectively.*

Rural discrepancy rates were significantly higher than urban. Error rates for both paper and tablet errors were also higher in the rural interviews, but these differences fell short of statistical significance (See Table 4).

**Table 4:** Paper-tablet discrepancies and device attributable coding errors with reference to residency status.

Discrepancies & Type of error	Urban (n = 47)		Rural (n = 48)		p-value
	Mean	%	Mean	%	
Discrepancies	9.40	4.02	12.83	5.48	0.0004
Paper errors	4.06	1.74	5.29	2.26	0.06
Tablet errors	4.21	1.80	5.08	2.17	0.12
Both paper and tablet errors	0.45	0.19	0.42	0.18	0.85
Unattributable errors	0.68	0.29	2.04	0.87	NA

*n: number of interviews.*

*%: Percentage of errors with reference to 234 comparison fields. p-value: Simple mixed binomial regression model with factor research assistant team.*

*NA: Not applicable.*

*The difference between the means of paper errors and tablet errors for urban and rural interviews is statistically insignificant. Paired t-test p-values for urban and rural setting are 0.83 and 0.75 respectively.*

The logistic model shows that device had no sizable effect on error-making, but the role of the field worker had a significant effect. (Table 5). The negative regression coefficient indicates that interviewers (L role) made fewer errors than observers who only recorded data (F role). Further stratification was done by role of the interviewer (L or F), to check for effect modification in the association between device and error-making, and this stratified analysis also showed no differences between interviewing devices (not shown in tables).

**Table 5:** Analysis of device and other determinants of paper and tablet coding errors: Mixed-effects binomial regression model.

Independent Variables	Estimates (*β)*	p-value†	95% CI
Tablet device (vs. Paper)	0.0068	**0.917**	[−0.122, 0.136]
Lead role (vs. Follower role)	−0.2599	0.000*	[−0.391, −0.129]
Interviewer_1	−0.8308	0.000	[−1.122, −0.538]
Interviewer_2	−0.3959	0.004	[−0.663, −0.128]
Interviewer_3	−0.7254	0.000*	[−0.964, −0.486]
Interviewer_4	−0.9441	0.000*	[−1.191, −0.696]
Interviewer_5	−0.6996	0.000*	[−0.932, −0.467]
Interviewer_6**	0	0	0

† *Wald test.*

*p-value<0.0001.

**serving as reference.

### Subjective preference and experience of interviewers

A total of 190 questionnaires, two each for 95 respondents, were completed–one each for the interviewer using the tablet device and paper form.. Paper forms were preferred in 18.75% of the interviews and tablet devices were preferred in 35.53%. Nearly half (45.72%) had no preference for either interviewing device (Table 6). Interviewers reported a few problems with both devices during the first 10 interviews, but none afterwards. Most of the reported problems were related to the place of interview, entering textual data in Marathi (*Roman characters*), difficulties reading screen under direct sunlight and some anxiety about working with costly tablet devices.

**Table 6:** Interviewer subjective preference for interviewing device.

Interviewer	Subjective preference			
	N	Paper (%)	Tablet (%)	No preference (%)
Interviewer-1	24	45.83	0.00	54.17
Interviewer-2	24	29.17	41.67	29.17
Interviewer-3	47	0.00	0.00	100.00
Interviewer-4	47	0.00	34.04	65.96
Interviewer-5	24	0.00	95.83	4.17
Interviewer-6	24	37.50	41.67	20.83
Mean percentage	190	18.75	35.53	45.72

*N = number of interviews.*

### Cost comparison

We had conducted 96 interviews with two tablets (Samsung Galaxy Note 10.1, Android OS). The cost of each tablet was USD 449, and server charges were estimated to be USD 50.40 for 24 weeks. The paper version contained 19 pages and the printing costs per interview were USD 0.70. Data double entry cost for a research assistant was estimated to be USD 9.26 per interview, assuming 30 minutes required for each interview. All these costs were converted from Indian Rupees to United States Dollars at a conversion rate of 1 USD for INR 54, as of January 2013.

For 96 interviews, the field study cost for paper interviews amounted to approximately USD 1,675 and for tablet interviews the amount was USD 1,700. Device-specific study cost with paper interviews amounted to USD 923 and USD 948 for the tablet. Additional costs for field operations not attributable to either device were excluded in the latter comparison. We had considered an optimistic scenario where the capacity of a tablet was assumed to be adequate for the first 400 interviews with reference to allotted time for the survey, but acknowledging a need for additional teams for each additional 200 interviews beyond that.

**Figure 1:**
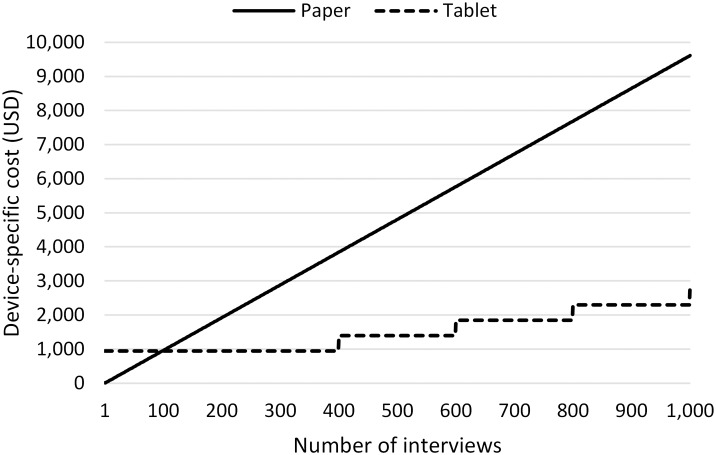
Device-specific cost comparison of tablet and paper studies based on sample size. The analysis assumes that costs of printing and data entry recur at a fixed rate for a paper interview study. It is assumed that for timely completion of the study, an additional team will be added for both paper and tablet interview studies of more than 400 interviews, and subsequently for further increases of 200. This imposes a device-specific additional cost for the tablet, but no additional device-specific cost for the paper interview study. Device-specific cost is equal for a study with n = 98. See text for additional assumptions on which this projection is based.

The projection for 1,000 interviews based on that assumption indicated a substantial shift in the balance of cost for paper- and tablet-based interviews. The cost for paper and tablet studies was approximately the same for a sample of 98 interviews. For larger studies, recurring device-specific costs for paper-based interviews gradually increased as the sample size increased, and device-specific costs for tablet-based interviews increased stepwise from 400 interviews increments of the cost of a tablet for each additional 200 interviews. This projection was based on planning for timely completion of the larger studies within a period of time that would not require additional cost for extended server time. The comparison of projected device-specific costs for tablet and paper studies is presented in figure 1.

Although there are no additional device-specific costs for an additional interview team, the total cost of the study would of course increase. Device-specific cost for the tablet team, however, will increase by the cost of an additional tablet device for that team. Our calculation does not include relatively small device-specific running costs, such as electricity to charge the tablets or to run the computers for data entry.

## Discussion

This experimental comparative study aimed to validate tablet devices for field-based epidemiological and public health research surveys. We developed an Android version of an EMIC interview for which we had experience using a paper form from a cultural epidemiological study in Pune, India. The study showed a low refusal rate among potential respondents, suggesting respondents in communities readily accepted tablet devices for household surveys. In the course of data cleaning for the paper interviews, we found notable differences between first- and second-entry data. This human-prone error-making at the data-entry step (2.49%) was totally eliminated for tablet-based interviews because there is no additional data entry step. This removes a potential source of errors, and it also saves the time and expense required for a subsequent data-entry step.

The identified discrepancies were analyzed to attribute errors to paper forms or tablet devices. The difference between paper errors and tablet errors was very low and far from reaching statistical significance. This suggests that both devices may achieve similar levels of accuracy in field-based research surveys. Among the three field-research assistant teams, the least experienced team (team C) made more mistakes with both paper and tablet. This shows less familiarity with the questionnaire leads to more errors with both devices. For the other two teams (teams A and B), there was little difference in error-making with paper but team B did considerably better with the tablet device. This suggests that teams with more experience and proficiency in the study design make fewer mistakes with tablet, even though no trend or learning curve was observed when we examined learning curves for discrepancies over the course of the study. Across urban and rural sites, there was little difference between devices for error-making. Within urban and rural sites also, there was no significant difference for error-making. The slightly higher error rates for paper and tablet devices in the rural site are likely explainable by the fact that the inexperienced team C worked only in the rural site.

The logistic regression model confirmed the absence of any sizable difference in error rates between the interview devices. The logistic model showed that the lead role was associated with fewer errors than the follower role. This may be explainable by a closer interactive relationship with the respondent, which ultimately helps in eliciting and recording data. It is also likely that routing questions according to response-specific skip logic may be more difficult to follow for the researcher in the F role (data entry only).

In nearly half of the interviews, researchers had no preference for either device, and more than a third preferred the tablet over paper. This showed that interviewers were comfortable using the tablet devices. There were a few problems reported in the first few interviews, but with more interview experience such problems were no longer reported. As the experience with the tablet devices increased, the field research assistants overcame initial problems. Technical problems were not an issue except for an inadvertent erasure of one record in an early interview.

The overall device-specific cost in this study for tablet-based interviews was slightly higher than for paper-based interviews due to the initial cost of the tablet device. This investment in the device will not recur, however, for future studies with that tablet. The same can be said for the cost of a computer purchased for data entry in a research study; our cost analysis regarded the data-entry computers, unlike the tablet devices, as an available resource of institutional infrastructure rather than an additional device-specific cost.

In a tablet-based study, device-specific costs are paid at the outset, and so the cost per interview becomes less with more interviews. Paper-interview studies may also have a non-recurring initial cost for storage, in addition to the recurring costs we considered for each interview for printing and data entry. Consequently, for large studies, the device-specific cost of using paper interviews becomes higher than for device-specific tablet-based interviews. In our projection of the cost for a study with 1,000 interviews, the device-specific interview costs were much less for a tablet study than a study with paper interviews. The difference was USD 6,866. We acknowledge, however, that this difference may be overstated because the printing cost per interview for printing a large number of interviews may be less than the figure based on 200 interviews that we used. Nevertheless, the cost benefit for the tablet is likely to remain substantial. We did not consider interviewer time in the cost comparison as the interview time was constrained to be identical for both instruments by the study design. Savings in the recurrent costs of the tablet instruments are potentially underestimated if the tablet enables more efficient interviewing that requires less time. We recognize that additional details in a more precise cost comparison may apply in specific settings. The cost of technical support, for example, depends on local expertise of the research team. Our analysis nevertheless provides a relevant approximation and guide for estimating costs of a study.

This experimental comparative study aimed to validate an approach for tablet-based data collection with regard to reliability and feasibility in the field. Successful XForm development on well configured Android tablet devices, well-trained interviewers, local engagement and a well-designed study protocol were the key factors for successful completion of this study. Elimination of the data-entry step with tablets did not result in increased error rates, underlining the high efficiency of this method. Similar findings have been reported in previous field-based research studies that used electronic devices, such as PDAs [3] mobile phones [4], [5] and tablet computers [7].

In a comparison study between the smartphone and paper-based interviews, the authors stated that the smartphones can be effectively used for implementing the data collection without sacrificing data quality and security [5]. In a review by Lane et al., the data accuracy with PDAs was found to be similar or better than with paper. They concluded that PDA-based data collection is less error-prone because of a more efficient process using range checks and systematic routing with skip logic. This point also applies to other electronic interview devices [2]. Shirima et al. argues that careful attention to applying experience from paper forms to electronic handheld devices will reduce human-prone data collection errors with electronic devices [20]. Yu et al and Shirima et al. [3], [20] had previously made the same argument based on experience with PDAs, and the point becomes more relevant as current devices have become much more sophisticated.

The cost comparison between the paper-based interviews and interviews with electronic devices was done in some studies, acknowledging the initial investment in electronic devices was higher than in the paper-based interviews [3], [5], [21]. Though the overall cost of tablet interviews was slightly higher than paper interviews in this study, the cost effectiveness of using a tablet can be achieved in large scale studies where the costs for data entry, cleaning and archiving paper forms become more substantial. Subsequent studies using previously acquired devices will of course cost less. Our findings from this analysis show that cost of the tablet device is not a serious barrier to its use. The cost of technical support, however, has not been considered in our analysis and may be more formidable, especially for research groups who have limited prior experience with the new technology.

There are very few studies reporting experience with qualitative data collection capacity of electronic devices. In a study by Zhang et al. on infant feeding practices in rural China, they collected open-ended answers with smartphones and data consisted of short text fields in Chinese characters [5]. In our study we collected some narratives in Marathi language with roman text using various entry options in tablets. The interviewers felt that typing or writing with handwriting character recognition available with the Samsung Galaxy tablet computer was not as easy as writing on paper, but they also felt that practice may improve writing speed and typing narratives. Additional prospects for use of swipe keyboard entry are also promising. Further research is needed to develop and test such options for their feasibility, and to enhance the capacity and value of tablets for working with qualitative data in text fields, and also for media (images, audio and video).

The loss of data in this study cannot be assigned to the device because ODK collect application has auto-saving and back-up options. Nevertheless, data for one record were lost because the interviewer deleted the whole form before finalizing it. One of the limiting factors for this study is the high profile required from interviewers. If a study has to be conducted with field workers with basic educational profile and less experience with technology, more time is required for training, and needs for technical support must be addressed. Updating knowledge and competence of researchers for use of new technologies becomes increasingly important to minimize needs and costs for technical support.

## Conclusion

Good acceptance by community respondents and clear preferences from interviewers, no higher error rates than with paper recording and similar data quality all show that the use of tablet devices is feasible, reliable and desirable for epidemiological and public health surveys. Using open source ODK software for Android devices, and advanced tablet hardware offers good prospects for efficient research without compromising data quality. Field research interviewers prefer tablet devices and respondents are comfortable being interviewed with them. Although the cost of tablet-based research requires initial investment in the devices, technical support may be a more formidable challenge than device costs. Over time and with larger studies, and with acquisition of technical expertise, tablet devices appear to be more cost effective than paper interviews. Our findings should motivate further development of capacity, competence and use of the new technology.

## Supporting Information

Data set S1 **Data which have been analyzed and presented in this report.**
(CSV)

Document S1 **Code sheet explaining the variables in the data set.**
(DOCX)
