# Supplementary material for: Developing and Validating a Tablet Version of an Illness Explanatory Model Interview for a Public Health Survey in Pune, India
Source: PLoS One. 2014 Sep 18;9(9):e107374. doi: 10.1371/journal.pone.0107374 (PMC4169412; doi:10.1371/journal.pone.0107374)
Supplement: Document S1 — Code sheet explaining the variables in the data set. (DOCX) [file pone.0107374.s002.docx]

Code book for discrepancy analysis- Mixed effects Binomial logistic regression model

| **Variable Name** | **Meaning** | **Value** |
| --- | --- | --- |
| EID | EMIC identification with setting followed by team and number of interview |  |
| LEAD | The leading role of the interview | TL/PL = 1  TF/PF = 0 |
| DEVICE | Interviewed device | Paper= 0  Tablet= 1 |
| SETTING | Interviewed area | Urban (U) = 1  Rural (R) = 0 |
| TEAMS | Assigned teams | Team A (A) = 1  Team B (B) = 2  Team C (C) = 3 |
| TeamA | AG/GT | Value of 1 to which the given team belongs to the respective interview |
| TeamB | MH/CD | Value of 1 to which the given team belongs to the respective interview |
| TeamC | MK/MG | Value of 1 to which the given team belongs to the respective interview |
| INTERVIEWER | Initials of the interviewer | AG = 1  GT = 2 CD = 3  MH = 4  MK = 5  MG = 6 |
| INTR_1 | AG | Value of 1 to which the given interviewer belongs to the respective interview |
| INTR_2 | GT | Value of 1 to which the given interviewer belongs to the respective interview |
| INTR_3 | CD | Value of 1 to which the given interviewer belongs to the respective interview |
| INTR_4 | MH | Value of 1 to which the given interviewer belongs to the respective interview |
| INTR_5 | MK | Value of 1 to which the given interviewer belongs to the respective interview |
| INTR_6 | MG | Value of 1 to which the given interviewer belongs to the respective interview |
| INT_SEX / SEX | Interviewer sex | Male = 0  Female = 1 |
| POI | Place of interview | 1: House hold  2: Field  98: Other (Ex. Temple, open areas and Shops etc.) |
| POI_CLS | Place of Interview (close or open space) | 1: Closed area  0: Open area |
| RES_SEX | Respondent sex | Male = 0  Female = 1 |
| RES_AGE | Respondent age | 18 – 65 |
| DOI | Date of interview |  |
| EDUE | Have respondent ever attended school or not | Yes: 1  No: 0 |
| MOS | Main occupational status of respondent | \| 1. Agriculture \| \| --- \| \| 1. Unskilled labour \| \| 1. Skilled labour \| \| 1. Self-employment (small business, petty trade) \| \| 1. Business (other) \| \| 1. Service (public sector) \| \| 1. Service (private sector) \| \| 1. Professional, specify___________________ \| \| 1. Student \| \| 1. Housewife \| \| 1. Retired \| \| 1. Unemployed \| \| 1. Other, specify_________________ \| \| 1. Cannot say \| |
| A_MOS | Adapted variable of MOS for analysis purpose | 1 = Employed (6,7) 2 = Self-employed (1,2,3,4,5,8) (98)  3 = Unemployed (9,10,12)  4 = Retired (11); 5= Cannot say |
| TVPP | Vaccine acceptors to prevent *Swine flu* | Yes: 1  No: 0 |
| DEN | Denominator | 234 |
| ERRORS | No. of errors (Outcome)  Sum of tablet errors, paper errors and both paper and tablet errors |  |
| T-P_Errors | Either tablet or paper error |  |
| T&P_Errors | Both tablet and paper errors |  |
| NS_Errors | Device non-specific errors |  |
| DISC | Total number of discrepancies per interview |  |
